# Molecular signatures of retinal ganglion cells revealed through single cell profiling

**DOI:** 10.1038/s41598-019-52215-4

**Published:** 2019-10-31

**Authors:** Lauren A. Laboissonniere, Jillian J. Goetz, Gregory M. Martin, Ran Bi, Terry J. S. Lund, Laura Ellson, Madison R. Lynch, Bailey Mooney, Hannah Wickham, Peng Liu, Gregory W. Schwartz, Jeffrey M. Trimarchi

**Affiliations:** 1 Present Address: Department of Molecular Genetics and Microbiology 2033 Mowry Road, University of Florida, Gainesville, FL 32610 USA; 2 Departments of Ophthalmology and Physiology, Feinberg School of Medicine Northwestern University, Chicago, IL 60611 USA; 3 Present Address: Oregon Health and Science University, Portland, OR 97239 USA; 4 Department of Statistics, 2117 Snedecor Hall, Iowa State University, Ames, IA 50011 USA; 5 Department of Genetics, Development and Cell Biology 2437 Pammel Drive, 2114 Molecular Biology, Iowa State University, Ames, IA 50011 USA; 6 Present Address: Emmune, Inc, 14155 U.S Highway 1, Juno Beach, FL 33408 USA

**Keywords:** Transcriptomics, Retina

## Abstract

Retinal ganglion cells can be classified into more than 40 distinct subtypes, whether by functional classification or transcriptomics. The examination of these subtypes in relation to their physiology, projection patterns, and circuitry would be greatly facilitated through the identification of specific molecular identifiers for the generation of transgenic mice. Advances in single cell transcriptomic profiling have enabled the identification of molecular signatures for cellular subtypes that are only rarely found. Therefore, we used single cell profiling combined with hierarchical clustering and correlate analyses to identify genes expressed in distinct populations of Parvalbumin-expressing cells and functionally classified RGCs. RGCs were manually isolated based either upon fluorescence or physiological distinction through cell-attached recordings. Microarray hybridization and RNA-Sequencing were employed for the characterization of transcriptomes and *in situ* hybridization was utilized to further characterize gene candidate expression. Gene candidates were identified based upon cluster correlation, as well as expression specificity within physiologically distinct classes of RGCs. Further, we identified *Prph, Ctxn3*, and *Prkcq* as potential candidates for ipRGC classification in the murine retina. The use of these genes, or one of the other newly identified subset markers, for the generation of a transgenic mouse would enable future studies of RGC-subtype specific function, wiring, and projection.

## Introduction

The vertebrate retina consists of distinct populations of cells and relies on 6 neuronal classes to work in harmony to respond to light from the environment in a way that will allow for reflex initiation, circadian photoentrainment, and image formation by the brain. These cell classes have distinct functions, with the retinal ganglion cells (RGCs) serving as the sole communication between retina and brain. There are more than 40 distinct subtypes of RGCs^1–3^ and the precise roles played by many of these cells in image formation are not fully understood. These cells can be classified morphologically and functionally based upon the specific information regarding visual stimuli that is transmitted between RGC to brain^1^. The purposes for varying classes of RGCs as well as the projection targets in the brain are unknown for many of these subtypes, and new RGC subtypes continue to be discovered. Attempting to uncover these functions would require knowledge regarding the transcriptome of a particular subtype for the generation of a vertebrate model possessing labeled or ablated cells of that subtype. Furthermore, the retina is an integral part of the central nervous system (CNS), which is comprised of thousands of different types of neurons^4^. The identification of markers for the classification of RGCs will likely extend beyond the retina and this tool may be useful for further characterization and identification of neurons in the entire CNS or may shed light on factors that label distinct classes of neurons throughout the CNS^5,6^.

Recent efforts to identify molecular signatures of rare and distinct cell types have relied heavily on single cell transcriptomics, particularly single cell RNA-Sequencing (scRNA-Seq). Many of these studies have employed this technique in the vertebrate retina, where cell populations and general wiring is well-studied and used as a model for the CNS in general. For example, the evaluation of murine bipolar cell markers has evolved from the use of microarray hybridization to RNA-Seq, allowing for a more in-depth examination of larger numbers of cells at a decreased cost^7,8^. More recently, scRNA-Seq has been combined with other techniques such as FAC-Sorting, imaging analyses, and electrophysiology to increase our knowledge base about the cells being examined, as well as to facilitate analysis and clustering. The application of scRNA-Seq extends beyond the retina and into various model organisms^9,10^, and has quickly become the favored technique for the discovery of novel cell type-specific molecular markers, especially for cells that are rare in a population. For this reason, we employed single cell transcriptomics to identify molecular markers of RGC subtypes in the mouse retina.

The identification of a molecular marker for each RGC subtype has been elusive to date, although groups have described genetic identifiers for some subsets of RGCs, including cocaine and amphetamine regulated transcript (*Cartpt*) for the four types of On-Off direction selective RGCs (ooDSGCs), homeobox D10 (*Hoxd10*) for the three types of On DSGCs and one type of ooDSGC, and melanopsin (*Opn4*) for the six types of intrinsically photosensitive RGCs, to name a few^1,11–13^. While these molecules have been useful in studies regarding limited populations of cells with similar functions, the specific function of each RGC subtype cannot be further studied without the ability to label these groups individually.

To identify markers of RGC subtypes, we employed three different approaches. First, we examined the transcriptomes of Parvalbumin (*Pvalb*) positive cells^14,15^. *Pvalb* has been observed in at least 8 subtypes of RGCs^16,17^, all of which project to the superior colliculus (SC) of the midbrain, the center of visual motor integration^17^. Much of the research involving the visual system has centered around lateral geniculate nucleus (LGN)-projecting RGCs, for their roles in image formation, though the SC is a major target of RGC axons^18^. Furthermore, 40 or so RGC subtypes have been characterized^3^, but more are estimated to exist^19^ and all of these subtypes lack distinct molecular markers^2^. We successfully identified many RGC subset markers and used hierarchical clustering analysis of the transcriptomes of these cells to reveal distinct populations of RGCs within the *Pvalb* + subset. Next, we employed electrophysiology to classify RGCs into their respective functional categories prior to examination of transcriptomes. Both approaches relied on hybridization to microarrays, thus enabling direct comparison of the resulting transcriptomes. Our final approach involved electrophysiological classification of RGCs prior to RNA-Sequencing in a similar manner to Patch-Seq^20,21^. Using *in situ* hybridization, several markers were validated due to their expression in various populations of cells among the mature mouse retina. These techniques allowed the identification of multiple genetic markers for distinct RGC subtypes which we expect will facilitate future in-depth studies of RGC subtype functionality, cortical projection, and intra-retinal wiring.

## Results

### RGC subset markers identified through transcriptomic analysis of tdTomato+ cells

*Pvalb* marks a subset of RGCs which remain largely uncharacterized at the transcriptomic level, so we set out to identify markers of these RGC subtypes by isolating *Pvalb*+ cells. We utilized offspring from a cross between the PV-Cre mouse line and Ai9 reporter mouse line, whereby tdTomato fluorescence was visible within those cells expressing *Pvalb*. Fourteen individual tdTomato+ cells were isolated and cDNA libraries were generated^22^. As an initial assessment of cDNA quality, samples were screened for the presence of a pan-RGC gene, synuclein gamma (*Sncg*)^23^ by PCR, as well as the absence of markers of possible contaminating cell types such as rod photoreceptors (Rhodopsin) and Müller glia (Glutamine synthetase). This assay, combined with agarose gel assessment of the cDNA smears, was used to determine the quality of our single cell cDNA libraries. Libraries with robust smears and favorable marker signatures were hybridized to Affymetrix Mouse 430 2.0 microarrays. Because *Pvalb* has also been observed in a minor population of ACs in addition to RGCs^24^, we began our full-transcriptome analysis by confirming the expression of a larger set of RGC-enriched genes. All 14 cells were found to express the RGC marker genes *Sncg*, neurofilament light (*Nefl*), and neurofilament medium (*Nefm*) (Supplementary Fig. 1). We compared the 14 tdTomato+ cells to several non-RGCs from previous publications to strengthen the identification of RGC-enriched genes^7,25,26^ and observed a greater amount of RGC-enriched genes among our isolated cells when contrasted by the non-RGCs (data not shown). Genes such as *Gap43, Ebf3*, and *Brn3b*^27^ were noticeably present among the tdTomato+ cells isolated (Supplementary Fig. 1).

**Figure 1:**
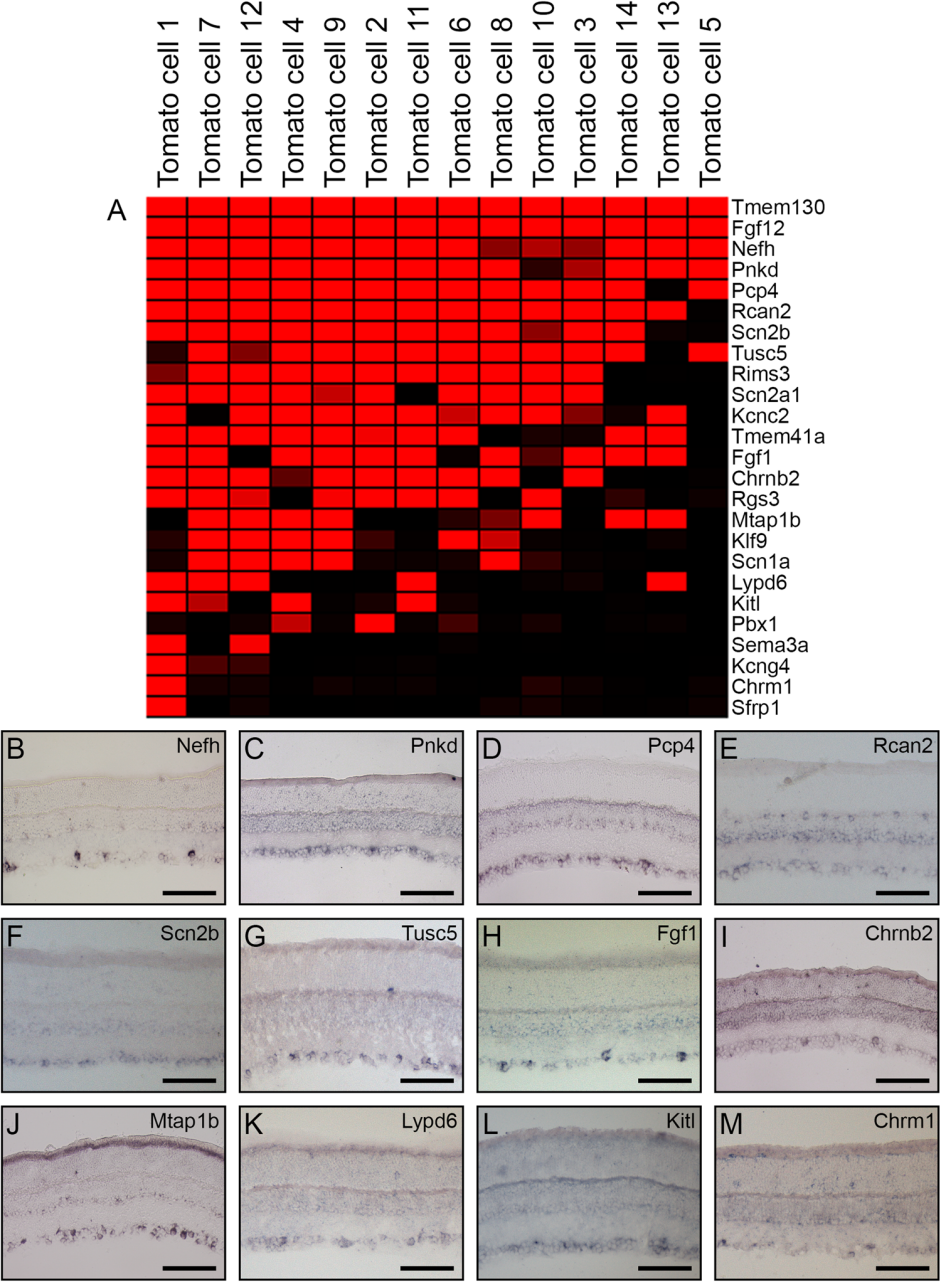
Retinal ganglion cell subset markers revealed through transcriptome profiling of tdTomato+ cells. Fourteen tdTomato+ cells were hybridized to Affymetrix microarrays and the resulting data was extracted and normalized by MAS5 software. The genes expressed in these cells were visualized on a heatmap created with Genesis software^75^, where red signal indicates high expression of the gene in a particular cell, and black signal indicates the absence of expression. Subset genes were identified based on their expression in the majority of the tdTomato+ cells (**A**) and were examined through *in situ* hybridization (**B**–**M**). Those examined include: *Nefh* (**B**), *Pnkd* (**C**), *Pcp4* (**D**), *Rcan2* (**E**), *Scn2b* (**F**), *Tusc5* (**G**), *Fgf1* (**H**), *Chrn2b* (**I**), *Mtap1b* (**J**), *Lypd6* (**K**), *Kitl* (**L**), and *Chrm1* (**M**). Scale bars represent 100 µm.

To assess the specificity of gene candidates among the total population of RGCs, we used the microarray data to identify potential marker genes and characterized expression patterns using *in situ* hybridization (ISH). First, we identified genes that were expressed among the broad class of RGCs based upon their expression within 7 or more cells. These genes were visually identified due to their expression among the majority of the 14 tdTomato+ cells (Fig. 1A), so we employed section ISH to investigate the expression patterns of eight of these genes and to assess their expression in the broad population of retinal neurons. In the adult retina, we detected expression within the GCL for all eight of these genes (Fig. 1B–I). *Nefh* was detected robustly in a subset of cells in the GCL and faintly in the INL (Fig. 1B), while *Pnkd, Pcp4*, and *Rcan2* were detected in a larger subset of cells in the GCL (Fig. 1C–E). Furthermore, *Pcp4* and *Rcan2* were also detected in the INL, expressed among a subset of ACs and HCs, respectively (Fig. 1D,E). *Scn2b, Tusc5, Fgf1*, and *Chrnb2* were all detected in a subset of cells in the GCL, with *Fgf1* and *Chrnb2* detected less robustly (Fig. 1F–I).

To assess the ability of our data to uncover factors expressed by subsets of RGCs, we initially performed a simple visual inspection of the transcriptomes of the tdTomato+ cells in an attempt to identify genes expressed by some, but not all, of our isolated cells. These factors were included in the study despite their lack of detection in the majority of isolated cells as we were interested to understand if the detection could reliably be correlated with expression in a subset of RGCs (Fig. 1A). We turned to ISH to investigate the expression pattern of some of these genes in more detail to determine if these subset candidates are expressed among smaller populations of RGCs by ISH and may therefore be valuable candidates for subtype markers. Through this examination, we uncovered four candidates for markers of limited RGC populations. *Mtap1b* was robustly detected in a subset of RGCs, which the other three genes, *Lypd6, Kitl*, and *Chrm1*, were all faintly detected in the GCL (Fig. 1J–M).

**Figure 2:**
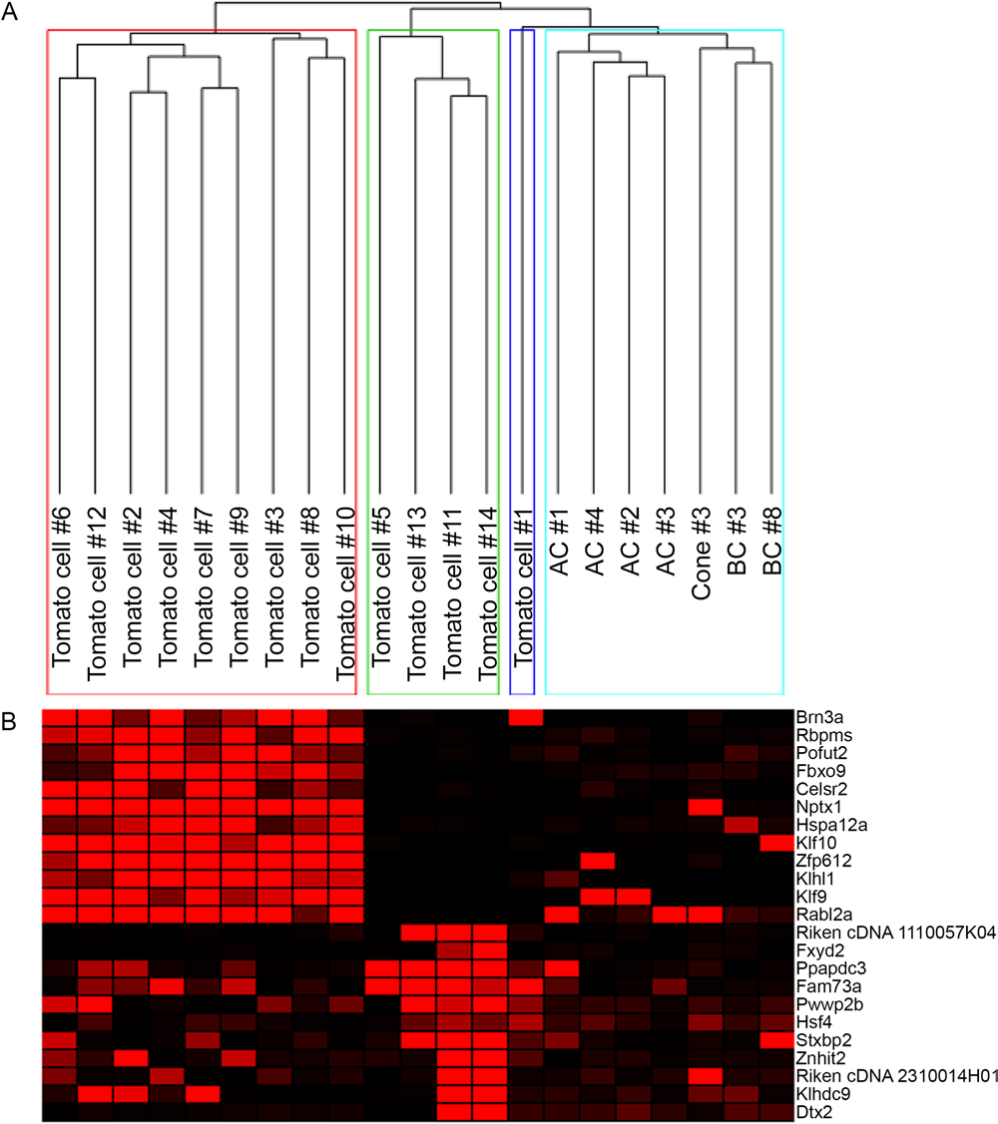
tdTomato cells cluster into distinct groups. The tdTomato+ cells were clustered using Pearson correlation with average linkage and 3 separate clusters of RGCs were identified (**A**). A heatmap showing the genes expressed highly by cells in those clusters (**B**), with red signal indicating high expression of the gene in a particular cell, and black signal indicating the absence of expression.

To identify markers of distinct RGC subsets, we examined clusters of the *Pvalb*+ cells using the entire dataset, rather than by tracking genes which correlated with one another. Through agglomerative hierarchical clustering, we identified 4 distinct clusters of cells, three of which contained the tdTomato+ isolates and one that contained bipolar cells (BCs), amacrine cells (ACs), and a cone photoreceptor that were included for comparison^7,25,26^ (Fig. 2A). Cluster 1 consisted of 9 different tdTomato cells, while cluster 2 contained 4 tdTomato cells (Fig. 2A). Cluster 3 consisted solely of tdTomato cell #1, which did not appear to be closely related to either of the two large RGC clusters (Fig. 2A), though it demonstrated some similarities in expression to cells in both clusters 1 and 2. Finally, cluster 4 contained the non-RGCs from previous studies, all of which clustered most closely together to cells of the same type (Fig. 2A). With this information in mind, we sought to identify molecular markers of the two main populations of tdTomato cells: clusters 1 and 2. The first cluster examined, cluster 1, was found to contain one gene previously identified as a subset marker: krüppel like factor 9 (*Klf9*), as well as *Klf10* (Fig. 2B). This cluster was also marked by the expression of *Brn3a* and *Rbpms*, two well-studied RGC markers. RGCs expressing the various BRN3 transcription factors have been examined for their distinct morphologies^28^ and associated transcripts have been identified through knock-out studies^29^. These factors have been shown to play crucial roles in development of the retina, as well as sustained presence in mature RGCs. Further, *Rbpms* has previously been observed in the retina as subset-specific among a class of RGCs^30^ and through correlation analysis, has been found to correlate highly with the BRN3 factors during development^29^, as we have observed here. The other factors observed in cluster 1 have not been investigated for their role in mature RGCs. However, *Nptx1* has been demonstrated to play a crucial role in synaptic development of the retina, where neuronal pentraxin proteins have been demonstrated to recruit AMPA receptors to synapses^31^. A recent study demonstrated the regulation of expression of *Nptx1* by *Brn3a*^32^, likely explaining our observation of the overlap between these two factors among RGC cluster 1. Next, cluster 2 was marked by the expression of factors including two Riken cDNAs, FXYD domain containing ion transport regulator 2 (*Fxyd2*), which has been observed in the human retina^33^, and approximately 8 other factors with high signal preferentially among cells in cluster 2 (Fig. 2B). We observed greater heterogeneity among cells of this cluster, suggesting to us that it likely is comprised of more than one subtype of RGC. Further, cluster 3 contained one tdTomato+ cell, which demonstrated expression of several factors detected by RGCs in both clusters 1 and 2. These results point to the uniformity of cells in cluster 1 and highlight the difficulty in relying upon clustering algorithms for small numbers of cells. Cluster 4 contained our non-RGCs, which successfully clustered together as we had expected: with the ACs forming a distinct tree from the BCs and the single cone photoreceptor.

During our observation of the 14 tdTomato+ cells we found that they did not easily fall into 8 separate categories as characterized by previous studies^16^. We were able to successfully identify one robust cluster of tdTomato+ RGCs and one smaller cluster which may be further refined with the addition of more cells. However, during these experiments we observed uneven fluorescence in this mouse line which likely led to greater selection of cells belonging to Cluster 1, rather than an even distribution of *Pvalb*+ RGCs. Specifically, since we could not use an antibody to amplify the reporter signal, it appeared that some RGCs expressed the reporter at higher levels and were therefore brighter and more easily isolated. Further, relying upon a reporter line to identify cells specific to a subset of the RGC population may result in the oversight of some cells which express the identifying factor at a low level, or those which fail to be labeled due to the nature of the transgenic model. An additional caveat for this approach exists within the nature of the transgenic model that was used, as the background of the PV^Cre^ mice is a segregating one. It is therefore possible that a retinal degeneration allele (*rd*8) is present in the background of these animals. However, in all of our experiments, we have never observed any perturbation to the ONL. Thus, characterizing a greater number of tdTomato+ cells may not allow us to achieve our goal and we felt it was best to adopt an alternative approach to classifying RGCs. We posited that the identification of genetic markers for RGC subtypes would be greatly facilitated by initially characterizing the cells into one of the many physiologically distinct subtypes.

### Electrophysiological classification of RGCs prior to transcriptomic analysis

**Figure 3:**
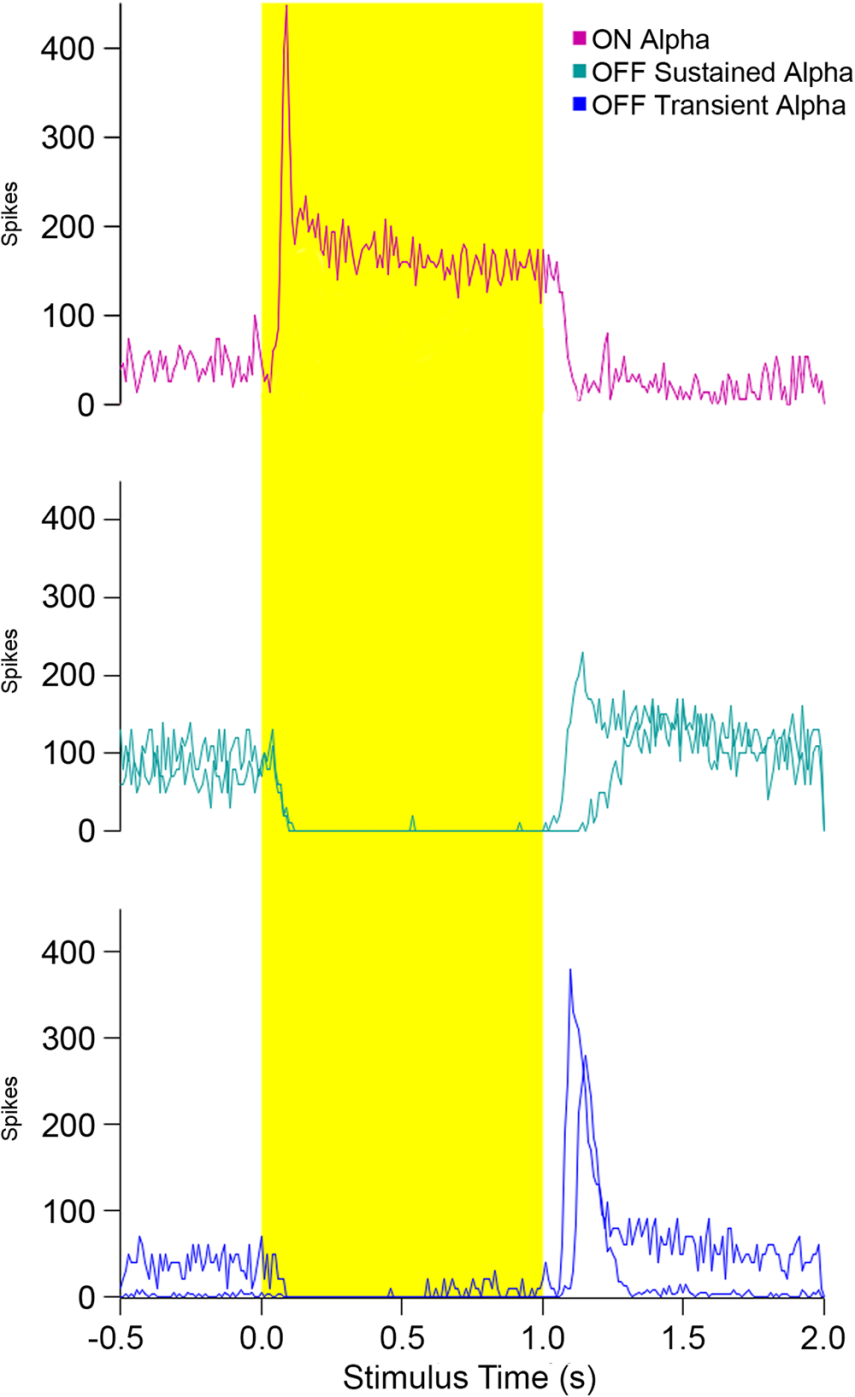
Spike trains of alpha RGCs following whole cell patch clamping. The spike trains of representative alpha RGCs were examined over a 2-second interval during which light was shone upon the cells’ receptive fields. On Alpha, Off Sustained Alpha, and Off Transient Alpha cells are shown.

In our second approach, cell-attached spike recordings were used to categorize mature RGCs into one of the 40 + previously identified functional subtypes. After this characterization, we isolated 29 cells, prepared cDNA libraries, and performed microarray hybridization to investigate their transcriptomes. RGC subtypes isolated included ON alpha^34,35^, OFF transient and sustained alpha^35–38^, ON direction-selective (DS)^12,39,40^, ON-OFF DS^39,41^, ON orientation-selective (OS)^42^, ON and OFF transient medium/small receptive field (RF), Pix_ON_^43^, J-RGCs^1^, local edge detectors (LED)^44,45^, and suppressed by contrast (SbC)^46^. The spike trains were plotted and used to classify each cell into its appropriate category, which included the distinct separation between ON and OFF responding RGCs, as well as those which maintain transient or sustained responses (Fig. 3).

To gain a general assessment of our second method, we sought to identify any common markers between the tdTomato+ cells and the categorized RGCs. We employed Pearson correlation to identify genes whose expression patterns were most closely correlated with the expression of *Pvalb* in both datasets (Supplementary Fig. 2A,B) and used these lists to identify factors commonly expressed in the categorized RGCs. These genes are enriched in our RGCs as compared to the non-RGCs and may be useful markers of this subset of cells. To demonstrate the approximate proportion of *Pvalb*+ cells in the retina, we used ISH to view the expression of *Pvalb* and pan-RGC gene *Sncg* (Supplementary Fig. 2C,D). Among both lists of *Pvalb-*correlated genes were known RGC markers, including stathmin 2 (*Stmn2), Brn3a/Pou4f1*, Thy-1 cell surface antigen (*Thy1*), and neurofilament medium (*Nefm*) (Supplementary Fig. 2). Using this data, we searched for novel RGC-specific genes among the *Pvalb* correlates. Two genes showed a high correlation with *Pvalb* in both datasets, annexin A6 (*Anxa6*) and cholinergic receptor nicotinic alpha 6 subunit (*Chrna6*) (Supplementary Fig. 2A,B). By ISH, *Anxa6* was expressed among a large population of RGCs, and *Chrna6* was expressed in a smaller subset of cells (Supplementary Fig. 2E,F). While *Chrna6* has been previously demonstrated as an RGC subset marker^47^, we demonstrate here the presence of *Anxa6* among a subset of these *Pvalb*+ cells, where it has not been characterized before. We also investigated the expression of four genes which were correlated with *Pvalb* in one dataset, but not the other. Among the tdTomato+ correlate list, we found potassium voltage-gated channel subfamily A member 6 (*Kcna6*) and protocadherin 7 (*Pcdh7*) expressed in a subset of RGCs by ISH (Supplementary Fig. 2G,H). From the characterized RGC correlate list, we investigated the expression of solute carrier family 6 member 17 (*Slc6a17*) and C-type lectin domain family 2 member L (*Clec2l*), both of which were detected in the GCL by ISH, though *Slc6a17* was also detected in the INL (Supplementary Fig. 2I,J). *Slc6a17* was more robustly and more broadly expressed, while *Clec2l* was detected in a subset of cells in the GCL, more akin to the expression pattern of *Pvalb* (Supplementary Fig. 2D,I,J).

### Identification of molecular markers for characterized RGC subtypes

**Figure 4:**
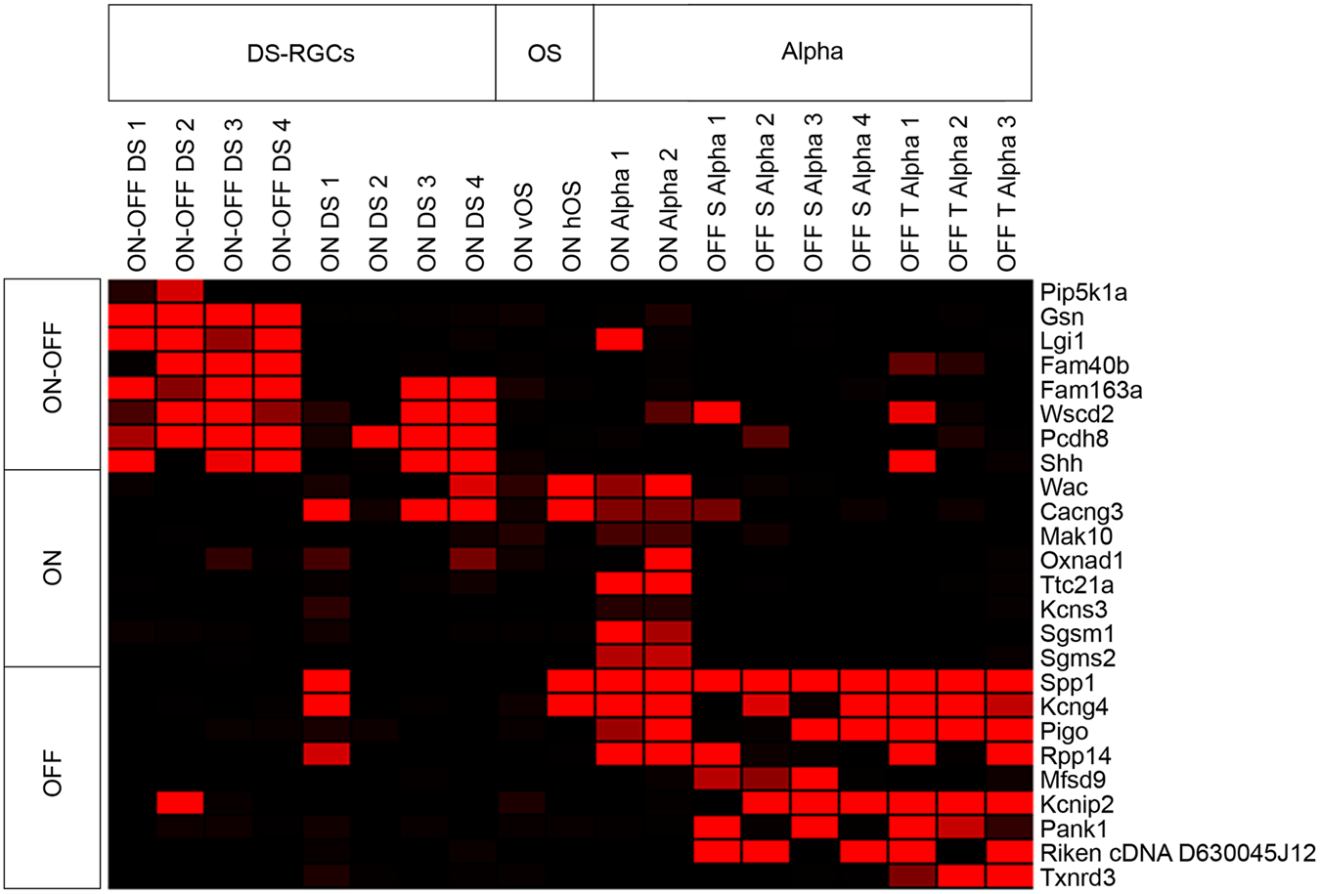
Markers of physiologically distinct RGCs identified. Three major groups of RGCs were classified: DS-RGCs, OS, and Alpha RGCs. The genes expressed by the cells belonging to these three major classifications are displayed on the heatmap. Clear distinctions were observed between cells with a preference for light during On-Off transitions, On alone, and Off alone.

Among these 29 isolated cells, 19 belonged to the DS (ON, ON-OFF), OS (ON), and alpha (ON, OFF) categorizations and therefore, were useful in the identification of genes expressed selectively among these broader subsets. We examined the enrichment of genes among these three subsets, and also characterized the expression of genes specifically within ON, OFF, and ON-OFF RGCs (Fig. 4) through visual examination of the transcriptomic data by identifying genes selectively expressed by members of the subsets and identifying highly correlated genes through Pearson correlation. Particularly, we observed the expression of genes such as family with sequence similarity 163 member A (*Fam163a*) and protocadherin 8 (*Pcdh8*) selectively among DS cells (Fig. 4). Markers of the alpha RGC subset included phosphatidylinositol glycan anchor biosynthesis class O (*Pigo*) and pantothenate kinase 1 (*Pank1*) (Fig. 4). In the distinction between cells which preferred ON, OFF, or ON-OFF stimuli, we observed a greater overlap between genes expressed by ON and ON-OFF preferring cells, while the OFF preferring cells appeared to have more distinct molecular signatures (Fig. 4).

The evaluation of transcription factor (TF) expression among RGC clusters has led to the identification of select combinations of TFs that allow for the differentiation of particular RGC subtypes^3^. One such combination was the co-expression of the MAF BZIP transcription factor B (*Mfab*) and potassium voltage-gated channel interacting protein 2 (*Kcnip2*) among a distinct set of RGCs. During segregation of the broad classes of functionally-classified RGCs, we observed the expression of *Kcnip2* by 6 of our 7 Off alpha RGCs (Fig. 4). We therefore decided to explore the overlap in expression of this TF and *Mfab*. Among the entire set of 29 isolated RGCs, we observed expression of *Kcnip2* and *Mfab* within 3 of the same cells (data not shown). Those cells belonged to one distinct subtype: transient Off alpha RGCs, and were not co-expressed in any of the other 26 cells, though expression of these two factors existed in varying proportions of these cells absent from the other factor. Thus, we suggest that cluster 39 from Rheaume *et al*.^3^ is likely enriched with a population of transient Off alpha RGCs.

We next decided to explore the expression of previously identified RGC subtype markers in our functionally distinct RGCs. The expression patterns of ooDSGC genes matrix metalloproteinase 17 (*Mmp17*), *Cartpt*, and *Jam2*, expressed by J-RGCs^11,48,49^, were observed. These genes were detected in overlapping expression patterns among our mature RGCs (Supplementary Fig. 3), though one would expect them to be expressed in separate populations, as the literature has demonstrated they should mark distinct subtypes of RGCs. Similarly, a separate analysis of known RGC markers and their expressions in clustered RGCs found very few of the published subtype-specific factors were restricted to a single subpopulation, though *Jam2* proved specific to J-RGCs in this study^3^. This finding suggests the expression of some previously characterized factors may not be as specific to the sole functional subtype to which they had previously been defined. Even though these previously identified markers did not track with a single subtype, we wished to identify genes that were strongly correlated with each marker, as these are still differentially expressed among RGCs even if they fail to track with a single subtype. We focused on correlated marker genes for *Mmp17, Cartpt*, and *Jam2* (Supplementary Fig. 3) and found approximately 10–15 correlated genes with comparable expression profiles for each factor, though none appeared to be confined to a functional subtype of RGC. *Mmp17* and ~5 of its correlates were detected in cells belonging to 5 distinct subtypes of RGCs (Supplementary Fig. 3). Within the *Cartpt* correlate list we observed protein phosphatase 3 catalytic subunit alpha (*Ppp3ca*), whose expression has been previously identified among a large population of RGCs in the chick retina by our group^50^ (Supplementary Fig. 3). The genes with most similar expression to *Cartpt* included *Mul1, BC023882, Col6a2, Sorl1, Mybph*, and *Syt16*, though none of these genes has been demonstrated to track with an individual RGC type. Finally, we observed *Jam2* expression in several of our isolated RGCs, and found genes such as *Scg2, Crbn, Mak10*, and *Jam3* highly correlating with the expression of this factor (Supplementary Fig. 3).

### *Opn4-*correlated genes among electrophysiologically distinct subtypes of RGCs

**Figure 5:**
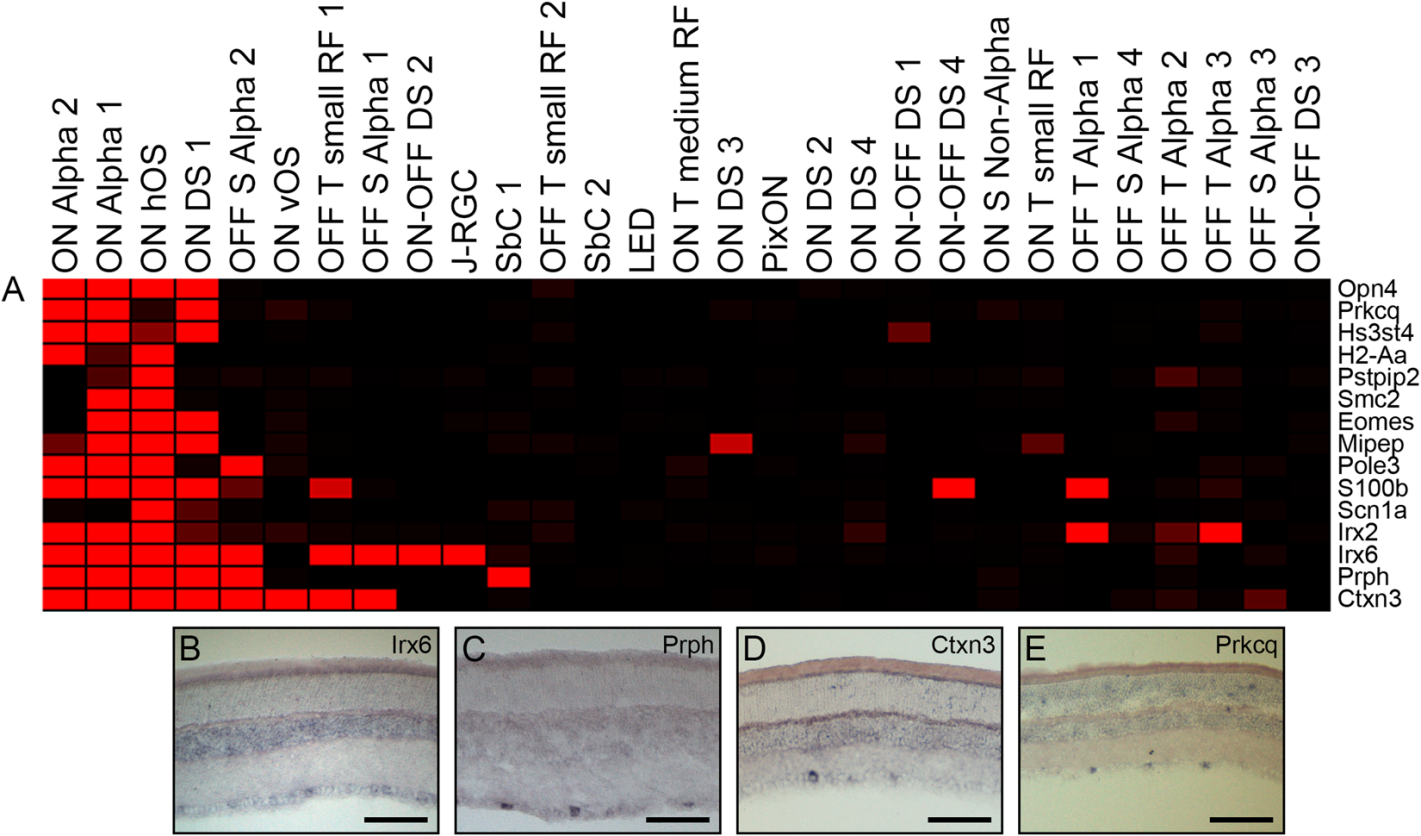
Potential genetic markers of ipRGCs. The expression of *Opn4-*correlated genes in the categorized RGCs was determined by Pearson correlation and visualized by heatmap (**A**). *Opn4*+ cell candidate markers were investigated by *in situ* hybridization (**B**–**E**). Genes examined included *Irx6* (**B**), *Prph* (**C**), *Ctxn3* (**D**), and *Prkcq* (**E**). Scale bars represent 100 µm.

We then examined genes correlated with *Opn4*, which codes for the melanopsin protein^51^ and is present only within intrinsically photosensitive RGCs (ipRGCs). The ipRGCs consist of 6 distinct subtypes of RGCs based upon their morphology, circuitry, and projection locations within the brain^52,53^. The functional profiles of these cells remain to be fully characterized, although the ON alpha RGCs have been identified as the M4 subtype^54,55^. In an attempt to identify markers of the ipRGC subtypes, we examined genes with high correlate scores (>0.53) in comparison to *Opn4* (Fig. 5A). Through this correlate analysis we detected a previously identified ipRGC gene, T-box brain 2 (*Eomes/Tbr2*^56,57^) within three of our *Opn4*+ cells, demonstrating the success of this analysis. We next explored some of these potential ipRGC candidates by ISH and found that Iroquois homeobox 6 (*Irx6*) was faintly detected in a subset of cells in the GCL (Fig. 5B). Three additional genes were promising candidates for ipRGC subset markers, as identified through this correlate analysis. Peripherin (*Prph*), cortexin 3 (*Ctxn3*), and protein kinase c theta (*Prkcq*) were all detected among a minute population of RGCs in the adult mouse retina by ISH, confirming their identification in less than 25% of our isolated cells by microarray hybridization (Fig. 5C–E). *Prkcq* was detected by microarray specifically within the our *Opn4*+ cells, suggesting this gene holds potential as a marker of ipRGCs, or even more selectively among one of the six ipRGC subtypes. Further, a recent publication which used clustering algorithms to evaluate the transcriptomes of over 6000 RGCs identified a cluster (#33 in Rheaume *et al*.) which was characterized by the presence of both *Prph* and *Ctxn3*, providing further evidence of our ability to detect subset-specific markers^3^. This cluster also contained S100 calcium binding protein B (*S100b*)^3^, which was detected among our *Pvalb*-correlate list (Fig. 5A). In looking more closely at the RGC subtypes gene browser, we observed the localization of *Opn4* expression in 4 clusters of Rheaume *et al*.: 6, 25, 26, and 33, with lesser detection of this ipRGC-specific factor among cells in clusters 37 and 21^3^. We next looked for the localization of our suggested subset factors in the RGC subtypes gene browser and were pleased to see a distinct overlap in clusters expressing *Opn4* and the three factors presented here. First, *Prkcq* was localized to clusters 6, 25, 26, and 33 – the same four clusters which demonstrated robust *Opn4* expression^3^. Next, *Prph* was detected in clusters 32, 33, and 39, while *Ctxn3* was found in clusters 6, 21, 25, and 26^3^. The data presented from this clustering analysis directly correlate with the findings observed in our study as *Prkcq* is expressed exclusively among our *Opn4*+ cells, while *Prph* and *Ctxn3* demonstrate a robust overlap among the cells which co-express these factors and *Opn4*, though both *Prph* and *Ctxn3* were detected in few *Opn4*- cells (Fig. 5A). We suggest that the data presented here, in conjunction with Rheaume *et al*.^3^, demonstrates the specificity of these noted factors and provides further evidence for their potential use as subset-specific markers.

### Ion channel subunits as candidate markers of subsets of RGCs

**Figure 6:**
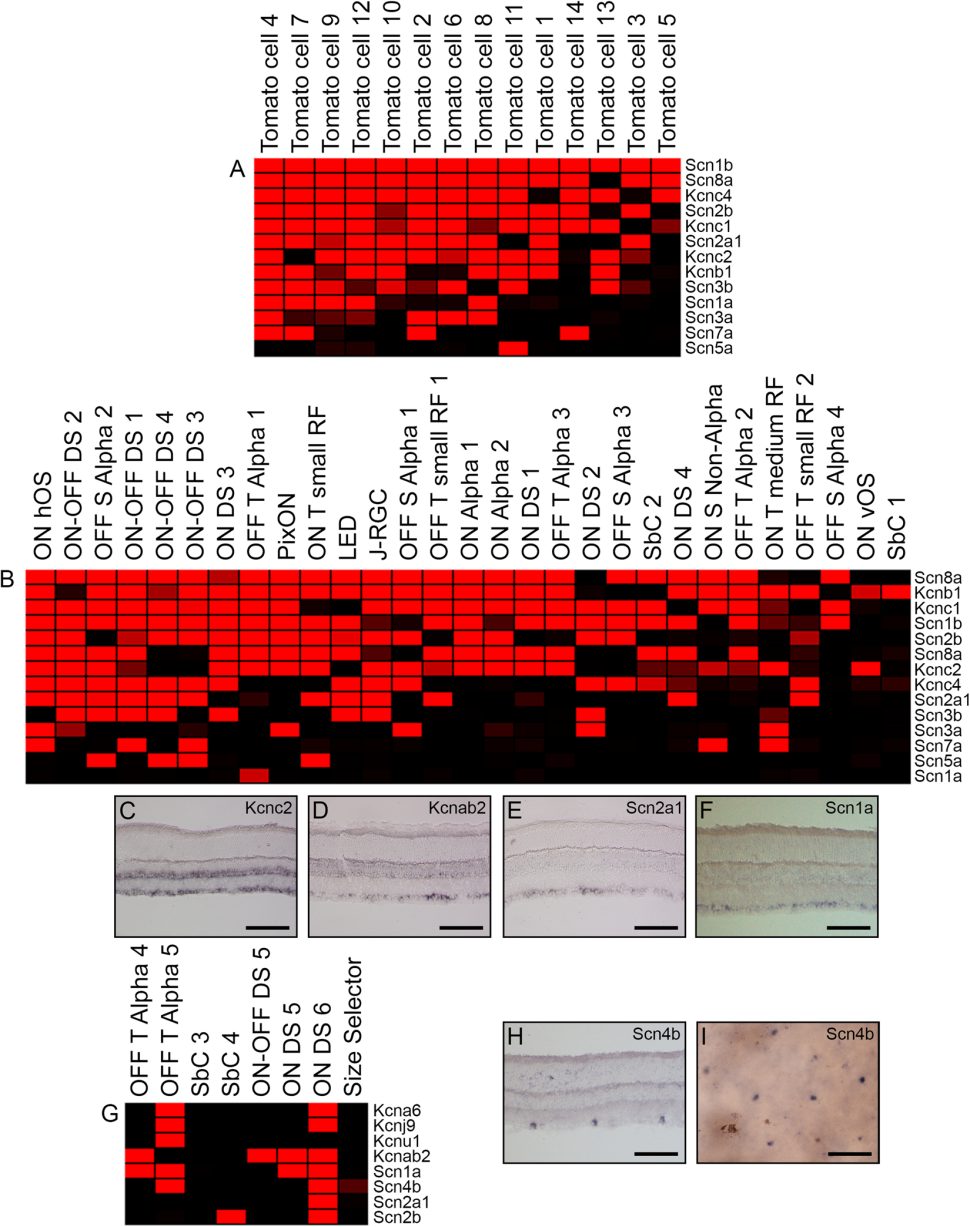
Voltage-gated channel genes detected in subsets of RGCs. The expression of potassium and sodium channel subunits were queried in our tdTomato+ (**A**) and physiologically characterized RGCs (**B**) and displayed by heatmap. Four of these ion channel genes were detected by ISH: *Kcnc2* (**C**)*, Kcnab2* (**D**)*, Scn2a1* (**E**), and *Scn1a* (**F**). Voltage-gated channel genes were then explored for expression among scRNA-seq data of 8 physiologically characterized cells and visualized on a heatmap (**G**). *Scn4b* expression was further examined by section ISH (**H**) and flat-mount ISH (**I**). Scale bars represent 100 µm.

Finally, during our search for potential subset markers of RGCs, we routinely observed voltage-gated ion channel genes within our correlate lists. Based upon previous studies which found *Kcng4* expression within both ON and OFF alpha RGCs^58^, we hypothesized that these channel genes might be good candidates for subtype markers. Therefore, we explored the expression of sodium channel and potassium channel subunit genes in both the tdTomato+ cells (Fig. 6A) and electrophysiologically classified RGCs (Fig. 6B). These subunit genes were highly enriched in our categorized RGCs, as compared to the non-RGC population, and their detection ranged from expression in all of the examined cells, such as *Scn1b* in tdTomato+ cells to expression in just one cell, as seen for *Scn5a* in the tdTomato+ cells and *Scn1a* in the classified RGCs (Fig. 6A,B). Due to this heterogeneous expression among the categorized cells, we employed ISH for eight of these genes, which we felt might be good RGC subset markers. Two potassium channel genes were examined: *Kcnc2* and *Kcnab2* as well as two sodium channel genes: *Scn2a1* and *Scn1a* (Fig. 6C–F). Both *Kcnc2* and *Kcnab2* were detected among RGC subsets, with detection of *Kcnc2* also in the INL (Fig. 6C,D). The sodium channel subunits were both confined to a subset of RGCs, with no detected expression outside of the GCL (Fig. 6E,F).

As part of a single cell RNA-Seq pilot experiment, we also isolated 8 functionally classified RGCs from the adult mouse and prepared these samples for RNA-Sequencing. These cells belonged to four broad RGCs classes: alpha, SbC, DS, and size selectors. We detected the expression of 4 potassium- and 4 sodium-channel genes within these 8 cells (Fig. 6G). One of these genes, sodium voltage-gated channel beta subunit 4 (*Scn4b*) was detected in very few RGCs by ISH (Fig. 6H). We therefore examined *Scn4b* by flat-mount ISH and detected expression among cells of the GCL in what appears to be a mosaic pattern (Fig. 6I). This subunit gene was among the most promising RGC subset candidate genes based upon its expression in so few cells of the adult retina. Future studies can utilize the genes discussed herein to generate transgenic mice for further characterization of RGC subtypes and to gain a better understanding of the roles these genes play in those distinct populations.

## Discussion

In the current study, we examined the transcriptomes of individual RGCs, with the ultimate goal of identifying genes expressed exclusively by different RGC subtypes. We employed the PV-Cre mouse due to the localization of tdTomato in eight RGC subtypes, therefore allowing for the sampling of distinct subtype transcriptomes on a lesser scale. The pursuit of subtype markers in a population containing eight subtypes seemed more straightforward than attempting to identify unique markers of each of the 40 + functionally characterized RGC subtypes. Further, previous attempts at classifying the eight subtypes of *Pvalb*+ RGCs based on their functional responses resulted in the observation that two of the eight types are direction selective, and three of the subtypes correspond to alpha RGCs in the mouse^1,17^. Therefore, single tdTomato+ cells were isolated and cDNA libraries were generated and hybridized to Affymetrix microarrays. We identified the expression of RGC-enriched genes in these cells, confirming their RGC character. We then explored the data for genes which were more broadly expressed throughout the isolated cells and employed ISH to characterize our candidate genes expression patterns in the sectioned tissue. While several factors were successfully identified in the GCL, others were also detected in the INL, possibly among displaced RGCs, though this may also suggest some of these factors were not RGC specific, but rather expressed by both RGCs and amacrine cells (ACs)-a characteristic of RGCs that has previously been described in the developing retina^27^ and appears to continue into adulthood.

While it was encouraging to identify previously characterized RGC markers among our tdTomato+ cells, we also uncovered several new markers of this subset that warranted further examination. *Pnkd, Pcp4, Rcan2, Scn2b*, and *Tusc5* were all detected among a subset of tdTomato+ cells and through ISH, the expression patterns of these genes were confirmed within a subset of cells in the GCL. We were intrigued to note the overlap in expression of *Pnkd* and *Tusc5* among the tdTomato+ cells as a recent study has suggested these two factors are regulated by *Brn3b* and *Brn3a*, respectively^32^. Our observation of their co-expression in the majority of our isolated cells demonstrates a potential regulation of these genes by other transcription factors. *Fgf1* has been previously examined for its importance in initiating the onset of RGC genesis in the chick retina^59^, and was found among a subset of tdTomato+ cells in our adult mouse retinas suggesting this growth factor plays a role in maintaining a subset of the RGC population. *Chrnb2* was detected in the GCL and among 10 of our tdTomato+ cells, indicating its potential as an RGC subset marker. Interestingly, previous examinations of knock out models for this gene found abnormal projection patterns of a subset of RGCs to the dLGN^60^, where these cells synapse with interneurons to relay information for image formation. While this study demonstrated the importance of this receptor during development, our studies indicate the continued expression of this gene within the adult retina. We detected the expression of *Mtap1b* among RGCs and observed its expression among a subset of tdTomato+ cells, showing a similar retention of this microtubule associated gene in the mature retina, where previous studies have identified its expression as crucial for the development of this tissue^61^. Finally, three factors were examined for their expression among a minor population of tdTomato+ cells: *Lypd6, Kitl*, and *Chrm1*. None of these genes had been characterized among RGCs in the adult retina prior to this study and were detected here in a subset of cells for the first time. Further, one of the factors identified as a potential subset marker, *Tusc5*, was identified in a separate study among a cluster of cells containing several uncharacterized factors, and was detected in approximately 10 of the 40 total clusters characterized^3^. Based upon the expression characterized here and by other groups, *Tusc5* demonstrates value as a RGC subset marker. We expect that this gene and its correlate may identify a distinct subset of cells in the murine retina, which have not been previously identified.

In an attempt to identify RGC subtype markers among the tdTomato+ cells, we employed Pearson correlation analysis with average linkage for the 14 RGC isolates and 7 non-RGCs. This analysis resulted in 3 distinct RGC clusters and 1 cluster containing the BCs, ACs, and cone photoreceptor. Only cluster 1 demonstrated clear patterns of expression distinction among tdTomato+ cells, suggesting more data is necessary to perform this type of analysis. Therefore, to use this *post hoc* clustering method to identify *Pvalb* + RGC subtypes, we would likely need many more isolated cells. We expect that an increased number of cells would allow for easier clustering and more distinct separation between the clusters of isolated cells. Further, a larger population of *Pvalb*+ cells would likely result in the identification of more clusters, assuming the tdTomato expression is equally represented among all *Pvalb*+ cells. However, we observed variation in the brightness of the tdTomato signal, so we expect the isolation of more *Pvalb*+ cells would likely lead to the isolation of more cluster 1 and 2 cells. Therefore, we hypothesized it would be useful to obtain more information about these cells prior to their isolation for facilitation of the identification of subtype markers and to utilize a non-transgenic strain to avoid any caveats regarding segregating the genetic backgrounds.

We employed cell-attached recordings of RGCs in the live retina prior to isolation and transcriptomic analysis for the procurement of subtype specific information for these cells. All of the 29 cells were classified functionally in a method similar to Patch-Seq.^20,21^ before the cells were isolated and hybridized to microarrays. Within this population of cells, eight belonged to the direction-selective subset, two to orientation-selective, and nine belonged to the alpha subset. We explored potential subset markers in this dataset and identified genes expressed by all three of these groups. At the same time, we were pleased to see clear genetic distinctions between cells classified as ON, OFF, and ON-OFF, where there was a noticeable overlap in expression between cells preferring ON and ON-OFF stimuli. We hypothesize this occurrence demonstrates a clear separation between OFF RGCs and all other RGCs, possibly occurring during development where these two populations may diverge early in retinogenesis.

We were intrigued by the similarities between the analysis of our tdTomato+ cells and the 29 functionally-characterized RGCs. For example, *Kcng4* and *Chrm1* were both detected in a single tdTomato + cell during our first analysis. Later, we observed an overlap in expression of *Kcng4* and *Kcnip2* among alpha cells during the observation of broad classes of RGCs. We feel even more confident in our detection of subset- and likely subtype-specific factors, as external groups have demonstrated similarities in clustering and expression analysis. For example, both *Chrm1* and *Kcnip2* were detected in a common cluster of RGCs by Rheaume *et al*.^3^, providing external validation of our ability to visually inspect and report accurately on the factors discussed herein. Further, as *Kcnip2* was detected within 6 of our 7 Off alpha RGCs, we propose the cluster identified is enriched with Off alpha cells. We expect that as more physiologically classified RGCs are profiled and paired with these two studies, more distinct markers of specific RGC subtypes will be identified.

One of the most intriguing findings observed in this study involved the exploration of a subset of RGCs. The ipRGC opsin gene, *Opn4*, was detected within four of our categorized cells, some of which are not previously characterized ipRGC subtypes. The detection of this gene within a potentially novel ipRGC subtype was intriguing. Four of the six ipRGC subtypes have been characterized morphologically; however, their specific light responses are not yet understood, though we have evidence that two of the remaining ipRGC subtypes are, in fact, previously characterized RGC subtypes. The ability to connect the dots between the functional and morphological RGC subtypes leads to a better overall picture of subtype diversity and also contributes to the convergence of information known about the current subtypes. We demonstrate here that ON DS RGCs and ON OS RGCs may be two of these previously unidentified ipRGC subtypes. While some of our ON DS and ON OS RGCs did not appear to express *Opn4*, it should be noted that the cardinal direction preferred by cells belonging to these classes serves as distinguishing traits and may be the reason why we see transcriptomic discrepancies between similarly subtyped RGCs.

Four genes were investigated by ISH, based upon their correlation with *Opn4* in our cells. Both *Irx6* and *Irx2* were highly correlated with *Opn4*, and *Irx6* was detected in a subset of RGCs by ISH, where it has been characterized previously^62^. The other three factors identified in this correlate analysis were of interest based on their expression in very few RGCs. *Prph* and *Ctxn3* were both detected in a subset of RGCs through ISH, neither of which have been previously identified as specific to a subset of mouse RGCs. However, a recent cluster-driven evaluation of a large population of RGCs identified a distinct cluster of cells expressing both *Prph* and *Ctxn3*^3^. Cluster #33 in this citation demonstrated increased expression of these genes as well as secreted phosphoprotein-1 (*Spp1*)^3^, a previously characterized marker of alpha RGCs^58,63^. On alpha RGCs are among the ipRGC population, known as M4, and thus may be the cluster identified in Rheaume *et al*., due to the expression of *Spp1* in this cluster paired with our observation of the correlation between *Prph* and *Ctxn3* with *Opn4*. Though not investigated by ISH, we also detected the presence of *S100b* as highly correlated with *Opn4*, and the presence of this gene was detected in cluster #33 of Rheaume *et al*.^3^. Furthermore, the identification of a cluster marked by both *Prph* and *Ctxn3* in this study of more than 6000 RGCs serves as external validation to our ability to successfully identify subset-specific markers. Finally, *Prkcq* expression was also investigated as this gene was highly correlated with *Opn4* and we detected expression of this factor in a subset of RGCs, which has been suggested previously^64^. Their detection within a distinct subset of RGCs demonstrates the potential for *Prph, Ctxn3*, and *Prkcq* to serve as molecular identifiers of distinct populations of RGCs, including the potential for these markers to selectively label the ipRGCs.

Our final effort to identify RGC subset markers arose from the detection of ion channel subunit genes in our datasets, including *Scn1a* among tdTomato + RGCs. Therefore, we decided to more thoroughly examine the presence of detected sodium- and potassium-channel subunit genes in our tdTomato + RGCs and electrophysiologically characterized RGCs. While other ion channels subunits were detected more broadly in our categorized RGCs, such as *Clcn5* in the *Jam2* correlate list, sodium and potassium channels were much more abundant across our cells and many of these channel subunits were detected in subsets of RGCs. Through ISH, we identified ion channel subunit genes which were expressed among a subset of RGCs: *Kcnc2, Kcnab2*, *Scn2a1* and *Scn1a. Kcnc2*, also referred to as K_v_3.2, has been demonstrated to play a crucial role in the regulation of fast-spiking RGCs in the rat retina^65^; we provide the first evidence for the expression of this gene among a subset of RGCs in the mouse. Na_v_1.2, the channel coded for by *Scn2a1*, was previously detected in several cell populations in the vertebrate retina of a later study^66^; however, our studies detected *Scn2a1* selectively among a population of cells in the GCL alone. The final gene in our voltage-gated subunit marker group was *Scn1a*. Though this sodium channel subunit gene has not been characterized among RGCs, its localization and function was deemed necessary for the normal function of parvalbumin-expressing interneurons of the mouse brain^67^. We hypothesize a similar mechanism among *Pvalb*+ cells of the retina, whereby *Scn1a* may add in the normal function of these cells. Interestingly, *Scn1a* was not present in all of our tdTomato+ cells, suggesting the possibility of a separate sodium channel subunit molecule among the remaining tdTomato+ cells.

Finally, we completed a pilot study of eight physiologically-classified RGCs from the mature mouse. The transcriptomes of these cells were examined via RNA-Sequencing for the presence of voltage-gated potassium and sodium channel genes, and 8 were found: *Kcna6, Kcnj9, Kcnu1, Kcnab2, Scn1a, Scn4b, Scn2a1*, and *Scn2b*. These genes were detected in varying amounts within our categorized cells and were detected through our microarray studies of RGCs, as well. One channel gene was detected in our pilot scRNA-seq study and evaluated for expression via ISH: *Scn4b*. We examined the expression of this gene in sectioned retinas, where it was detected in a minor population of cells in the GCL; therefore, we considered the potential for this gene to be present among a physiologically distinct subtype. To test this hypothesis, we utilized flat-mount ISH for the examination of *Scn4b* + mosaicism in the GCL. The presence of RGC subtypes in the GCL has been characterized in a mosaic arrangement, where the cells of a single subtype are expected to evenly “tile” the surface of the retina^68^. With this in mind, we set out to examine whether *Scn4b* would be detected in a mosaic pattern among RGCs, which it was. This finding points to the potential for *Scn4b* to be a genetic marker of a functionally distinct subtype of RGCs in the mouse and warrants further investigation. Comparing this study with others that have utilized clustering algorithms to segregate large quantities of RGCs, we can begin to glean information about the distinct genetic profiles of functionally characterized RGCs. The analyses discussed here lay the groundwork for future studies of single cell molecular identifiers, particularly of RGCs and demonstrate the effectiveness of electrophysiological characterization of cells married with transcriptomic profiling.

## Methods

### Ethics statement

All procedures for the care and housing of mice conform to the U.S. Public Health Service Policy on the Humane Care and Use of Laboratory Animals and were approved by the Institutional Animal Care and Use Committee at Iowa State University and Northwestern University School of Medicine.

### Single cell isolation

#### Manual isolation from PV;Td-Tomato mouse

To generate the Pvalb-Tomato line, the PV^Cre/+^ mouse was obtained from the Jackson Laboratory (Jax) (*Pvalb*^*tm1(cre)Arbr*^ Jax 008069^14^) and crossed with the Ai9 Cre reporter line (*Gt(ROSA)26Sor*^*tm9(CAG-tdTomato)Hze*^ Jax 007909^15^). The PV^Cre^ line originated on a C57BL/6;129P2OlaHsd segregating genetic background, while the Ai9 reporter exists on a congenic C57BL/6J background. Since the PV^cre^ line originated on a segregating background, we cannot 100% rule out the presence of a retinal degeneration allele. However, mice from these crosses did not exhibit retinal degeneration in our lab and that was consistent with results observed by other research groups^69–71^. Specifically, eyes from mice derived from this cross were routinely cryosectioned and observed via ISH and immunohistochemistry between 4 weeks and 6 months of age. In all of these experiments, we never observed any photoreceptor degeneration phenotype as the ONL was intact. These two strains were interbred within our facility and were maintained and used only as double heterozygotes for both alleles. The mice resulting from this breeding scheme had visible Td-Tomato within those cells that expressed parvalbumin. Tissue dissociation and cell isolation was carried out as described^22^.

#### Manual isolation following electrophysiological categorization

Retinas from C57BL/6J mice were dissected under IR light (940 nm) and cuts were made along cardinal directions before the retina was mounted, GCL up, on a 12 mm coated glass coverslip (BioCoat Cellware, Corning). The coverslip was secured to the recording dish and placed on the electrophysiology rig (SliceScope Pro 6000, Scientifica, UK) and the retina was superfused with 32 °C pre-warmed carbogenated Ames medium (US Biological Life Sciences). Tissue was illuminated at 950 nm for visualization and cell attached recordings were completed using pipettes filled with Ames solution via 2-channel patch-clamp amplifier (MultiClamp 700B, Molecular Devices). To display visual stimuli, a custom-designed light projection device (DLP LightCrafter, Texas Instruments) was employed and experiments relied on blue LED illumination with a peak spectral output at 450 nm. Various visual stimuli were projected upon the retina and the response of each RGC was monitored as previously described^72^.

Once a given RGC was classified, a newly fire-polished glass electrode was used to aspirate the soma and transfer it to a nuclease-free PCR tube for molecular processing. Isolated cells to be hybridized to microarray chips were placed in lysis buffer and immediately processed, while cells to be used for RNA-Sequencing were expelled into TCL Buffer (Qiagen) containing 1% β-mercaptoethanol (Sigma), briefly spun on a tabletop microcentrifuge, and immediately frozen at −80 °C for up to two weeks before processing.

### Microarray hybridization sample preparation

Cells in lysis buffer were processed as previously described for hybridization to Affymetrix microarrays^22^. cDNA libraries were analyzed by agarose gel electrophoresis to assess the quality of the library. Those samples with a smear from 300bp-1Kb were then prepared for microarray hybridization by biotinylation^22,27^. Hybridization of samples to Affymetrix 430 2.0 Mouse Genome Arrays was carried out at the Iowa State and University of Iowa DNA Facilities, and normalization and transformation of the resulting data was performed using the MAS5 algorithm. The files for the tdTomato+ cells have been deposited in the Gene Expression Omnibus (GEO) (GSE115332) and for the electrophysiologically defined cells (GSE115379).

### RNA-Sequencing sample preparation

Single cells were stored at −80 °C for no more than 2 weeks before samples were processed. Cells were thawed to room temperature (RT) for 1 minute, then incubated with Agencourt RNAClean XP beads (Beckman Coulter). After a brief incubation on a magnetic separator device, the supernatant was removed and RNA was washed thrice with 70% ethanol. RNA was briefly air dried before rehydration in water. Reverse transcription was carried out using the Smart-Seq v4 Ultra Low Input RNA Kit (Clontech), with minor modifications to manufacturer’s instructions. Briefly, reaction buffer and 3’ SMART-Seq CDS Primer II A were added to samples and incubated at 72 °C for 3 minutes. Reverse transcription was then carried out through the use of Ultra Low First-Strand Buffer, SMART-Seq v4 Oligonucleotide, RNase inhibitor, and SMARTscribe Reverse Transcriptase. The reaction took place as follows: 42 °C for 90 minutes, 70 °C for 10 minutes. Immediately following reverse transcription, cDNA was amplified using a cocktail of SeqAmp PCR Buffer, PCR Primer II A, and SeqAmp DNA Polymerase. The PCR program was used as per manufacturer’s instructions, though the number of cycles was increased to 34. cDNA libraries were purified through the addition of Agencourt AMPure XP Beads. Following incubation on a magnetic separator device, supernatant was removed and DNA was washed twice with 70% ethanol. DNA was resuspended in elution buffer and quality was assessed on the 2100 Bioanalyzer (Agilent Technologies). Samples were then tagmented using the Nextera XT Low Input kit (Clontech). 0.5 ng of cDNA was combined with TD buffer and TDE1, then incubated at 55 °C for 5 minutes. Following the addition of buffers NT1 and NT3, supernatant was discarded and NT3 was added a second time. Supernatant was again discarded and resuspension buffer was added to DNA. Purified DNA was isolated and moved to a new tube before indexes were added. Unique combinations of indexes were added to samples with NPM and PPC and briefly amplified using the following program: 72 °C for 3 minutes, 96 °C for 30 seconds, 8 cycles of: 98 °C for 10 seconds, 63 °C for 30 seconds, and 72 °C for 3 minutes. DNA beads were again employed for purification of DNA samples, which were ultimately rehydrated in resuspension buffer. Samples were pooled and sequenced on the Illumina 2500, with an average read length of 100 bp at the Iowa State University DNA Facility. Files for the RNA-Sequencing data have been deposited in the Sequence Read Archive (SRA) at NCBI and the SRA accession number is PRJNA548506.

### Cluster analysis

The 14 tdTomato+ cells were clustered using agglomerative hierarchical clustering^73^ wherein each cell begins as a cluster onto itself. To cluster cells most effectively, many genes were filtered out of the data set to reduce the background noise and facilitate clustering of the 14 tdTomato+ cells and 7 non-RGCs. First, the standard deviations for all 45,101 microarray probesets within RGCs or non-RGCs were examined using histograms (Supplementary Fig. 4). A mixture of two normal distributions fit the distribution of standard deviations well. Genes belonging to the normal distribution with a smaller mean were considered not to be differentially expressed, but rather were likely to contribute to the noise of cluster analysis. Therefore, we retained genes if their standard deviations were above 2.5 for both the RGCs and non-RGCs. This resulted in a final dataset consisting of 8,037 probesets for the clustering analysis. Clustering was carried out with Pearson correlation and average linkage. Cutting the tree into 4 groups resulted in three tdTomato + clusters and one non-RGC cluster.

### *In situ* hybridization

Probe sequences between the sizes of 650–850 bp were amplified from mouse retina cDNA using the primers listed in Table 1 and cloned into pGEM-T (Promega).

**Table 1:** Primer sequences for generation of ISH riboprobes.

Gene	Primer 1	Primer 2
*Anxa6*	ctgtgcacccgtagctatcc	agtcaggcagggttatgtgg
*Chrm1*	tggacagcccagagagactt	gtgggaccgaggtcacttta
*Chrna6*	cgaagctcctgctggttatt	ctcccatttgggttgctcta
*Chrnb2*	agcaccagtgttggttttcc	agggtcctgtgtcctgtttg
*Clec2l*	tatctcgggtatgggggaat	gataggacagcaccacagca
*Ctxn3*	tctggattccctggacgat	cctaccactgcttcctggag
*Fgf1*	cccccacatatggacaagac	gcctggatggatactcagga
*Irx6*	ggaagacctggaggaagagg	gtgtgtgtcatctggcctgt
*Kcna6*	ccaatctgggatgtcatggt	tccgtctcagtcactgcttg
*Kcnab2*	caccctagctttgccttctg	ctggccttacaccctttgag
*Kcnc2*	ttttgggtatgagaaggaag	tctgatgtctgtggcgtctc
*Kitl*	gagcccttatgccacacaat	ggatcactcctaagcccaca
*Lypd6*	gctgtcatttccagcctagc	tgtggttgtggaaggagaca
*Mtap1b*	gtcccagtcctgaggagaag	gacacgtcatctgtggttgg
*Nefh*	gccaccaagggagagaagta	cagaagcacttggttttattgc
*Pcdh7*	ttctctggtgggtcttttgg	tttctgtgtcatgccagctc
*Pcp4*	ggtgaatgcctctcattggt	tcgctcatcttacctccttttt
*Pnkd*	cccacacttcaccatcctct	ctgaggcagaccacagttca
*Prkcq*	tcctggaatctctcacagca	caaatagcatgcatgggttg
*Prph*	acatccgtgcacagtacgag	aggctgagatcagggtcaga
*Pvalb*	ggatgtcgatgacagacgtg	tgcagagattgaacgaggtg
*Rcan2*	ggactgttccggacctatga	cacaggactgaactggagca
*Scn1a*	caatgtccacagcagcttgt	gacaaacccagctcagcaaa
*Scn2a1*	gaggcttctgttttcgcaac	agtcatgctgcctggactct
*Scn2b*	tgctaattaagggccactgc	gctgggtcacagaagaccat
*Scn4b*	agtgggctacagcacctctc	gccagaggactaaaccatgc
*Slc6a17*	attcggaagctgacctgaga	gcatgtggagaggaggagag
*Sncg*	cagtccatagcttgcagcag	cacagcagcatctgattggt
*Tusc5*	tcctcgttttctgtgggaag	gagtcactagtgcgtgtggtg

The sequences of primers used in the generation of gene-specific RNA riboprobes for ISH are included here.

Probes were synthesized and *in situ* hybridization was carried out on adult mouse retinal cryosections as previously described^50^. Images were acquired on a Nikon Eclipse 55i microscope. Adobe Photoshop was used to crop and lighten photos so as to minimize background signal; no other manipulations were performed.

#### Tissue preparation and probe hybridization in whole-mount

Whole-mount *in situ* hybridization was performed on adult mouse retinas as previously described with some modifications^74^. Briefly, eyes were removed from adult mice, immediately fixed with 2% PFA in 1X PBS for 15 minutes and transferred to 2X PBS for 5 minutes. Retinas were dissected in 2X PBS and flattened in an empty Petri dish, GCL up, before ice-cold methanol was slowly dripped on the retinas until they were white and rigid. Retinas could be stored at −20 °C for up to 2 months at this stage. For *in situ* hybridization, retinas were incubated in 4% PFA for 5 minutes before two 5-minute washes in PBS + 0.1% Tween-20 (PBT). The tissue was partially digested for 15 minutes in digestion solution (proteinase K [1 μg/ml], 6% SDS, 0.25% Tween-20) and post-fixed in 4% PFA for 4 minutes. Retinas were then washed twice for 10 minutes in PBT and transferred to a six-well plate containing PBT. Formamide wash buffer (20X SSC, 0.1% Tween-20, 50% formamide) was added between the wells of the six-well plate, as well as within the empty wells. The PBT surrounding the retinas was removed, hybridization buffer was added, and retinas were incubated at 65 °C for 10 minutes, or until the retinas had adhered to the plate. Before hybridization, the digoxigenin-labeled probe (~700–800 bases) was incubated at 95 °C for 5 minutes in hybridization solution. Following adhesion of the retinas to the plate, the probe solution was then added, and the plate was tightly sealed before incubation at 65 °C overnight. The next morning, retinas were washed at 65 °C in formamide buffer once for 10 minutes, then twice for 20 minutes. They were washed twice for 20 minutes with TNT at RT and then blocked in 5% HISS/TNT for 20 minutes. The α-DIG-AP antibody was subsequently added in blocking buffer and incubated at 4 °C overnight.

On the final day, retinas were washed in TNT once for 5 minutes, twice for 25 minutes, and 3 times for 45 minutes at RT. Retinas were incubated in alkaline tris buffer (1 M Tris pH9.5, 1 M MgCl_2_, 5 M NaCl, 0.1 Tween-20) for 10 minutes at RT and staining was performed in alkaline tris buffer containing NBT and BCIP for up to 3 hours at 37 °C. Once the desired staining was achieved, retinas were washed twice for 1 hour in alkaline tris buffer, then mounted in glycerol between two cover slips. Retinas were imaged on a Nikon Eclipse 55i microscope. Adobe Photoshop was used to crop and lighten photos and minimize background signal; no other manipulations were performed.

## Supplementary information

Supplemental Figures
