# Supplementary material for: Molecular signatures of retinal ganglion cells revealed through single cell profiling
Source: Sci Rep. 2019 Oct 31;9:15778. doi: 10.1038/s41598-019-52215-4 (PMC6823391; doi:10.1038/s41598-019-52215-4)
Supplement: Supplementary file 1 — Supplemental Figures [file 41598_2019_52215_MOESM1_ESM.pdf]

**Molecular signatures of retinal ganglion cells revealed through single cell profiling**

Lauren A. Laboissonniere[1], Jillian J. Goetz[2], Gregory M. Martin[3], Ran Bi[4], Terry J.S. Lund[5], Laura Ellson[5], Madison R. Lynch[5], Bailey Mooney[5], Hannah Wickham[5], Peng Liu[4], Gregory W. Schwartz[2], and Jeffrey M. Trimarchi[6]*

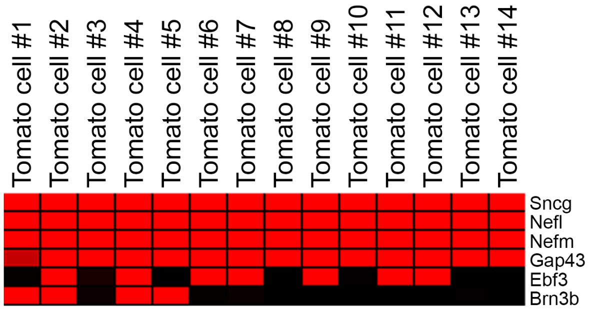


**Supplementary Figure 1. RGC marker genes expressed in tdTomato+ cells.**
The expression of RGC genes was examined in our tdTomato+ cells and visualized by heatmap. Genes examined included *Sncg, Nefl, Nefm, Gap43, Ebf3,* and *Brn3b.*

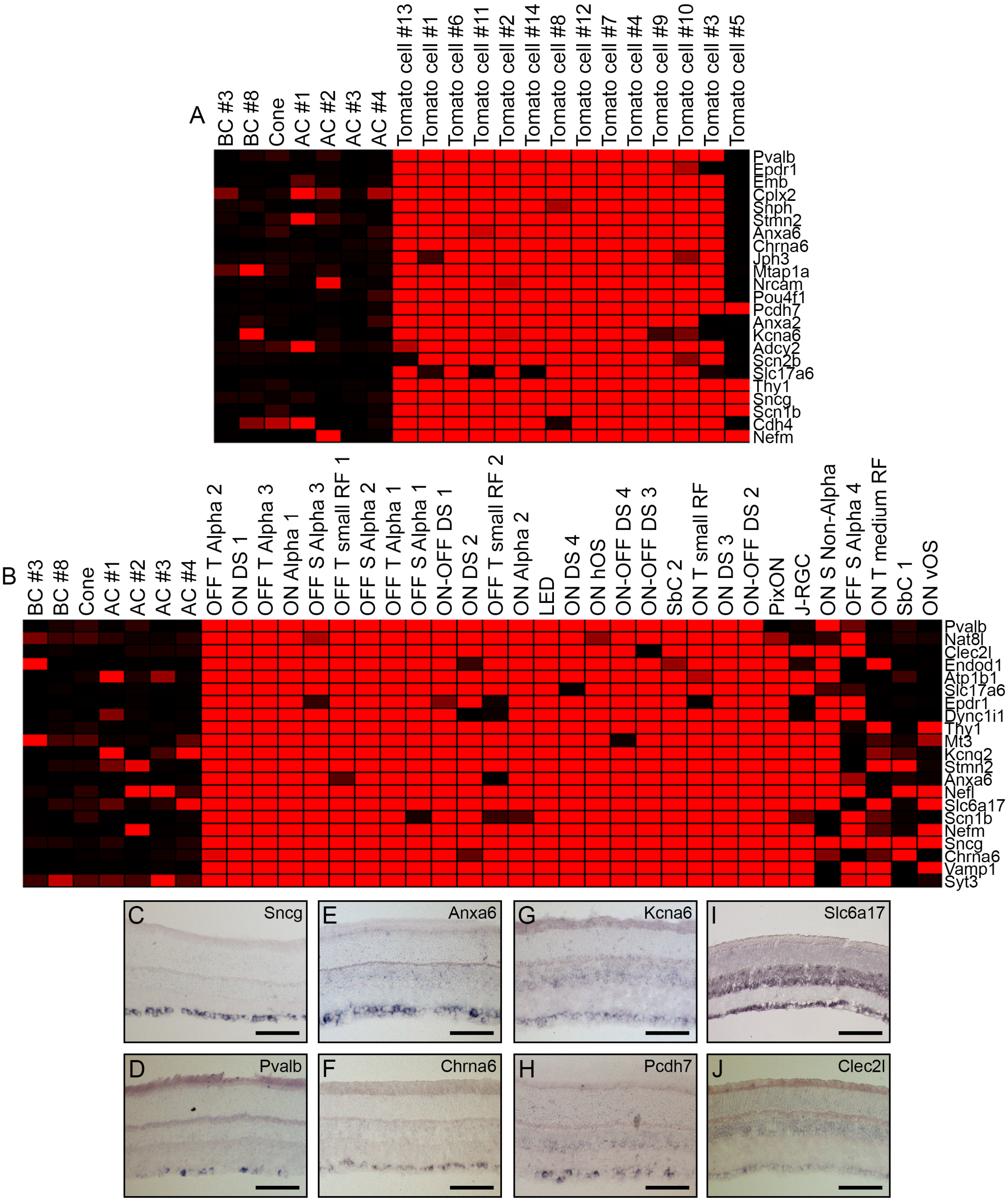


**Supplementary Figure 2. Parvalbumin gene correlates detected in RGCs.**
Genes highly correlated with *Pvalb* in the tdTomato+ cell population were determined through Pearson correlation and visualized by heatmap (A). Among the physiologically classified RGCs, *Pvalb* correlated genes were also defined by Pearson correlation and demonstrated through heatmap (B). *Pvalb* correlated genes from both datasets were then examined by ISH: *Sncg* (C), *Pvalb* (D), *Anxa6* (E), *Chrna6* (F), *Kcna6* (G), *Pcdh7* (H), *Slc6a17* (I), and *Clec2l* (J). Scale bars represent 100 μm.

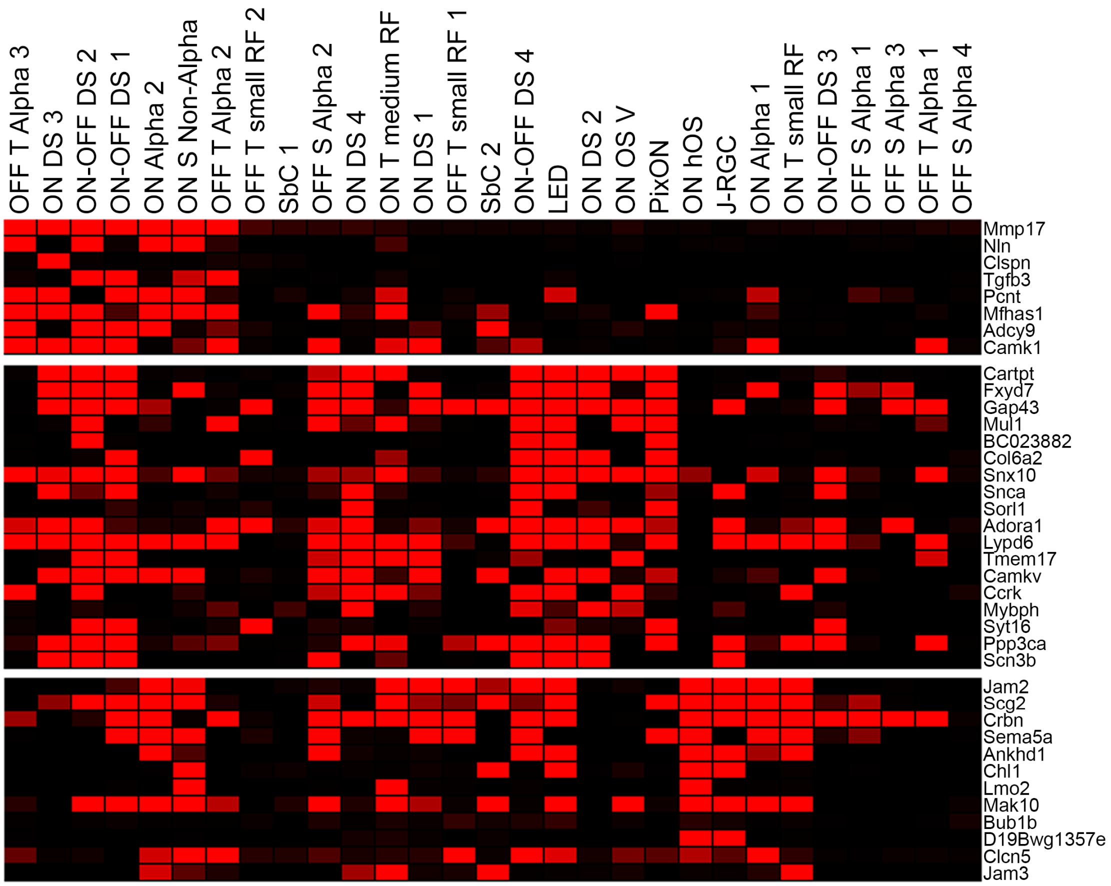


**Supplementary Figure 3. Genes correlated with RGC subtype markers.**

The genes which were most highly correlated (Pearson correlation) with previously identified RGC subtype markers, *Mmp17, Cartpt,* and *Jam2*, were identified within the characterized RGCs.

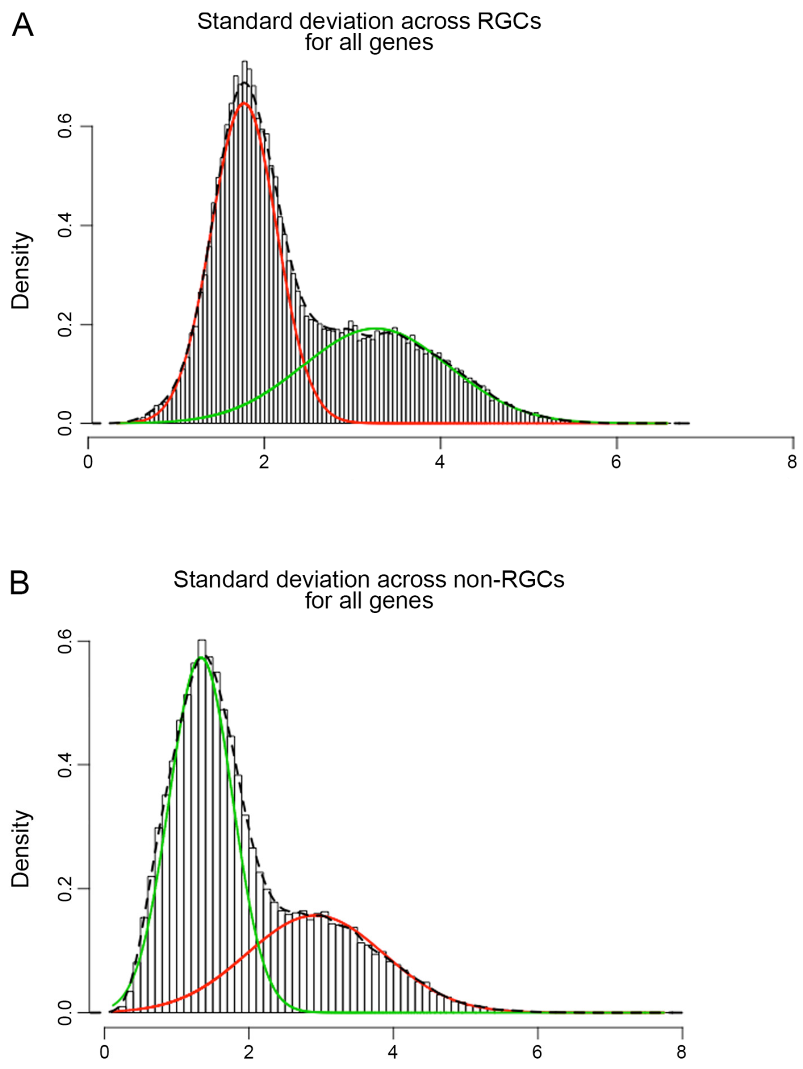


**Supplementary Figure 4. Standard deviation of microarray data across RGCs and non-RGCs.** The standard deviation of gene expression among the physiologically characterized RGCs (A) and non-RGCs (B) was examined.
